# Interprofessional Education at Colleges of Osteopathic Medicine Improve Confidence in Interprofessional Teamwork and Patient Handoffs

**DOI:** 10.7759/cureus.106077

**Published:** 2026-03-29

**Authors:** Stefanie J Driesenga, Rebecca G Burrington, Ankit Jain, Sara Selinsky, Raafeh A Waseem, Christopher B Divito

**Affiliations:** 1 College of Medicine, Lake Erie College of Osteopathic Medicine, Greensburg, USA

**Keywords:** aacom, coca, interdisciplinary cooperation, interprofessional education, patient safety improvement

## Abstract

Patient safety is a cornerstone of medical practice, and teamwork is critical in creating a culture of safety. Training osteopathic medical students in interprofessional communication is hypothesized to improve confidence in coordination and effective transitions of responsibility within the healthcare team. The Commission on Osteopathic College Accreditation adopted Interprofessional Education Collaborative goals in 2019. Several studies have assessed attitudes towards teamwork and patient safety at the level of a hospital unit or team. However, there are few studies analyzing college of osteopathic medicine student confidence levels over multiple campuses in reference to specific Interprofessional Education Collaborative goals.

Our study retrospectively analyzed student responses to the American Association of Colleges of Osteopathic Medicine Graduating Seniors Survey. Our analysis showed that participation in interprofessional education was positively correlated with increased confidence in performing safe and effective patient handoff from one healthcare team to another and confidence in working as a member of a healthcare team. Additionally, we identified specific interprofessional educational activities that correlate with increasing confidence in specific Interprofessional Education Collaborative goals. This study indicates that interprofessional education is a vital component of effective medical school curricula, but some training activities are more effective than others in building student confidence.

## Introduction

Safety is an area of major concern in medical practice. Medical errors are a significant cause of disability, death, and healthcare costs in the United States [1]. Contributors to medical errors are numerous, and establishing a culture of safety within a healthcare system involves all members of the healthcare team [2]. Additionally, the cost of safety failures extends beyond the patient and their family and can have a significant negative psychological impact on those healthcare team members involved [1]. Therefore, teamwork and communication are paramount to ensure patient safety and proper healthcare operations.

The Joint Commission's National Patient Safety Goals identified failure points to patient safety which include incorrectly passing on patient medicines to the next provider during patient handoffs, failure to follow standard processes within a system, and other communication errors [3]. A retrospective review of a random sample of 50 malpractice claims from a national claims database identified communication failures in 48% of claims [4]. In 47% of those claims, provider-provider miscommunication was identified, and 40% of the claims involved failed patient handoff. To improve communication and teamwork in healthcare, the Interprofessional Education Collaborative (IPEC) was formed in 2009 by several health professional educational organizations with the goal of enhancing team-based learning and improving patient safety outcomes. In 2011, the IPEC released core competencies for interprofessional care, with the most recent version released in 2023 [5]. The goals of the IPEC core competencies are to promote interdisciplinary, team-based patient care and improve population health. As of 2023, the collaborative represents 22 health professions. Weaver et al. reviewed the success of team and communication training and reported that 80% of trainee groups reported increased confidence in patient safety [2]. Therefore, interprofessional team training is an essential element in improving patient safety and health outcomes.

In 2019, the Commission on Osteopathic College Accreditation (COCA) added an Interprofessional Education for Collaborative Practice standard for accreditation [6]. Accredited colleges must ensure that their curriculum includes interprofessional training and these standards closely mirror IPEC's core competencies. These educational goals are accomplished by providing learning experiences in academic and/or clinical environments that permit interaction with students enrolled in other health professions degree programs or other health professionals. Therefore, to improve patient safety, interprofessional team training and education are now a required curricular component of osteopathic medical schools.

Background

Starting with the 2012-2013 year, the American Association of Colleges of Osteopathic Medicine (AACOM) surveyed graduating seniors to determine interprofessional education (IPE) participation in line with the goals of the IPEC core competencies and COCA standards. AACOM surveys include measures of student confidence felt in various aspects of medical practice, including during patient handoffs and the ability to participate as a contributing and integrated member of an interprofessional team [7,8]. However, there are few reports investigating the use of IPE in United States medical schools. Jones et al. commented on the lack of evidence for the effectiveness of IPE; however, their data was consistent with osteopathic (DO) students being better positioned to engage in interprofessional learning than the allopathic (MD) students [9]. In a study by Carpenter et al., IPEC core competency training through simulation and faculty feedback improved College of Medicine (COM) student confidence in patient-family communication [10]. Moreover, there have been several studies demonstrating improvements in attitudes of students who utilized IPE [11]. In addition to addressing communication and patient safety, the introduction of IPE in earlier years could circumvent the development of any bias toward other health professionals [12]. However, the optimal time to introduce IPE within the medical school curriculum has not been standardized as educational strategies for IPE delivery and assessments of outcomes vary across medical school programs [13]. Indeed, the lack of standardization may lead to varying degrees of student learning outcomes and confidence in participation as an interprofessional team member. Therefore, suboptimal IPE training could lead to greater patient risk and poorer health outcomes.

The objective of this study was to evaluate the association between IPE participation and osteopathic medical student confidence in patient handoffs and interprofessional teamwork and to identify which specific IPE activities are most strongly associated with increased confidence. In this study, we retrospectively analyzed the AACOM Graduating Senior Survey data collected from 2014 to 2022 to investigate these relationships [7]. In this study, we demonstrate that IPE in COM are associated with increased confidence in patient handoffs and the ability to work as part of an interprofessional team. This work also provides valuable insight into what IPE activities were most impactful on self-reported student confidence. 

## Materials and methods

This study is a retrospective analysis of repeated cross-sectional survey data collected from graduating osteopathic medical student seniors through the AACOM Graduating Seniors Survey [7]. The de-identified data was directly obtained, with permission, from the AACOM. This study was considered exempt by the Lake Erie College of Osteopathic Medicine Institutional Review Board (IRB) under protocol number 31-035.

Student responses to the questions (i) "What kinds of educational experiences did you have with other health professions students?", (ii) "How confident are you in your current ability to perform the following activities: Perform safe and effective transitions of responsibility for patient care from one healthcare team or practitioner to another?" (patient handoffs), and (iii) "How confident are you in your current ability to perform the following activities: Participate as a contributing and integrated member of an interprofessional team and fully embrace the value of teamwork in patient care?" (teamwork) on the AACOM survey were investigated. Students were allowed to respond to the question "What kinds of educational experiences did you have with other health professions students?" by indicating any number of the following activities: "Lecture (Basic Science)", "Lecture (Clinical Subject)", "Patient-Centered Case Studies", "Clinical Simulations", "Active Engagement with Patients (Rotations of Any Kind, Clinics)", "Community Projects or Service Learning", and "Skills Training in Team Setting Workshops". Student self-reported confidence measures for patient handoffs and teams were limited to a five-point Likert scale with "Not at all competent", "Slightly incompetent", "Neither competent nor incompetent", "Slightly competent", and "Very competent".

Normalized confidence levels indicate the five-point Likert scales converted to a numerical score from -2 to +2 to analyze the data where positive values indicated increased confidence and negative values indicated decreased confidence. Data points were excluded from this report where graduates did not respond whether they took part in IPE or not, gave incomplete confidence ratings, or were unsure of IPE participation. Finally, we excluded responses for the year 2023 as the survey measures were discordant compared to previous years. Additionally, the AACOM raw survey data used the term "competence level"; however, the published survey summary report uses the term "confidence level" [7]. In this study, we equated the two terms. Data were analyzed using Python (Version 3.11.5, packaged by Anaconda, Inc., Austin, Texas, United States) in Jupyter Notebook (Version 7.0.8), and figures were plotted using Prism (Version 10, GraphPad Software, Boston, Massachusetts, United States).

## Results

**Figure 1:**
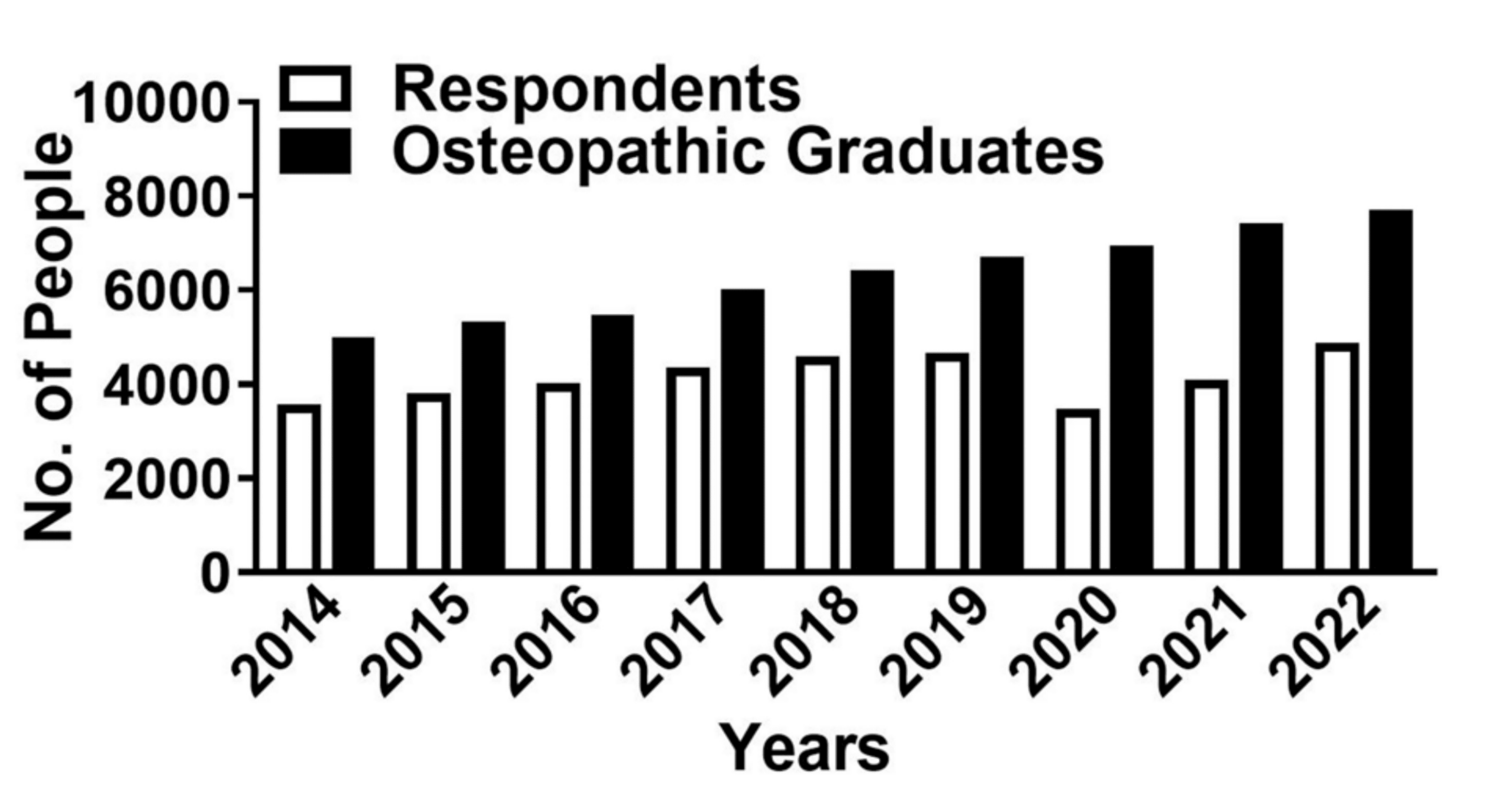
Student graduations and survey responses Bar graph detailing the number of osteopathic medical student graduates in the United States (black bars) and the number of AACOM Graduating Senior Survey respondents (white bars) between 2014 and 2022. AACOM: American Association of Colleges of Osteopathic Medicine

The AACOM graduating senior survey data from 2014 to 2022 was analyzed for self-reported student participation in IPE activities. The number of student respondents who participated in IPE training increased from 3,569 to 4,868 from 2014 to 2022, a 26.7% increase. However, the number of graduating seniors in that timeframe increased from 4997 to 7702, a 35.1% increase, indicating a net decrease in response rates of approximately 8% (Figure 1).

**Figure 2:**
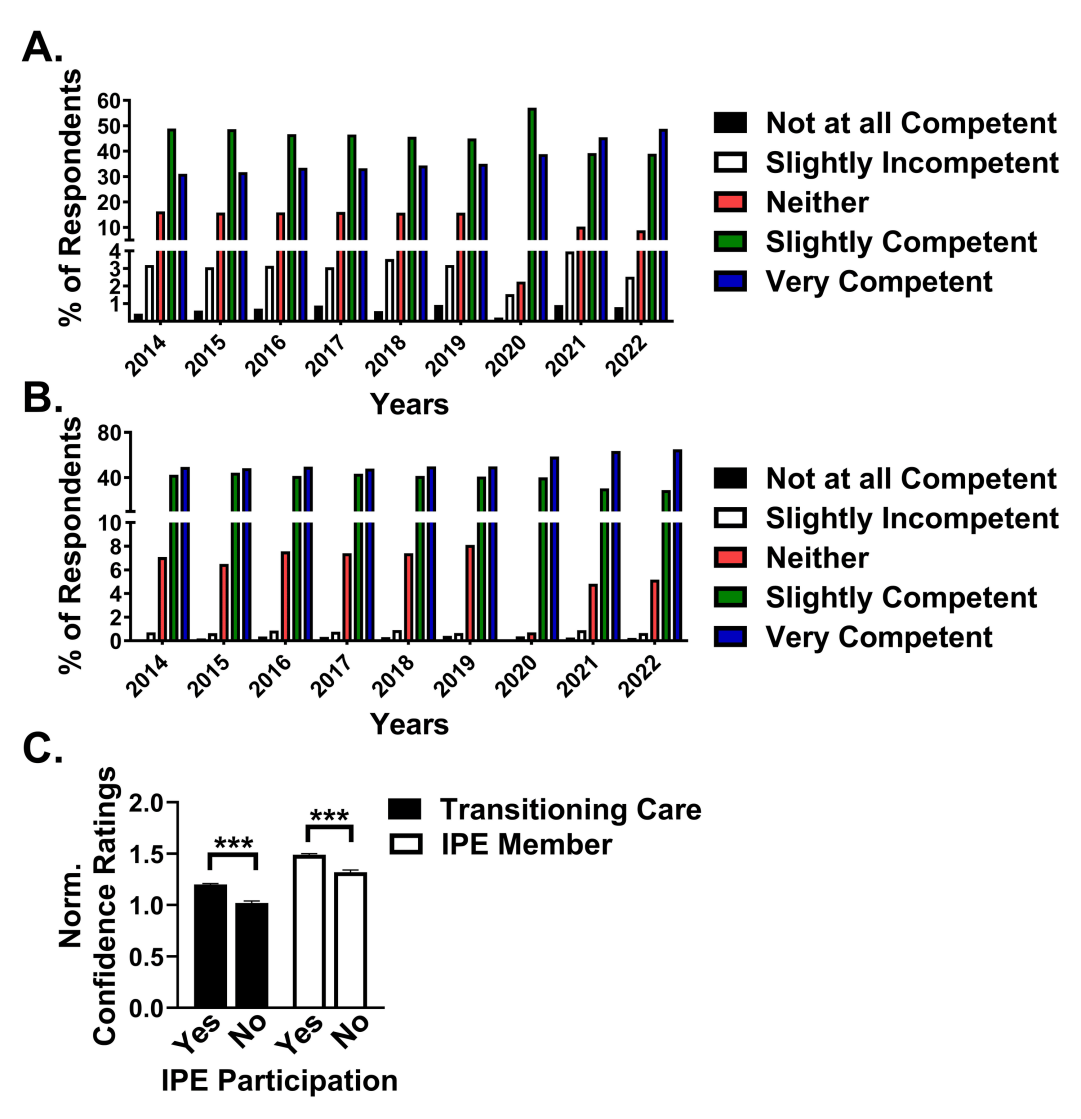
Confidence measures in patient handoff and teamwork (A-B) Bar graph depicting the percent of respondents who reported "Not at all competent" (black bars), "Slightly incompetent" (white bars), "Neither competent nor incompetent" (Neither; red bars), "Slightly competent" (green bars), or "Very competent" (blue bars) for (A) five-point Likert scale responses to the question "Rate your confidence in your ability to: Perform safe and effective transitions of responsibility for patient care from one healthcare team member or practitioner to another" and (B) five-point Likert scale responses to the question "Rate your confidence in your ability to: Participate as a contributing and integrated member of an interprofessional team and fully embrace the value of teamwork in patient care". (C) Bar graph of normalized, average Likert scale average responses for both patient handoff (black bars) and team member (white bars) confidence separated by student response to participation in IPE activities. *** indicates a p-value of <0.001. IPE: interprofessional education

To determine how IPE training participation affected self-reported confidence, two measures were analyzed using a five-point Likert scale ("1": "Not at all competent"; "5": "Very competent"). The first measure analyzed was self-reported confidence to "Perform safe and effective transitions of responsibility for patient care from one healthcare team member or practitioner to another" (patient handoff). The respondents who reported "Very competent" increased from 1,089 out of a total 3,499 respondents (31.12%) in 2014 to 2,317 out of a total 4,749 respondents (48.79%) in 2022 (Figure 2A). Interestingly, respondents who reported "Not at all competent" also increased from 15 (0.43%) in 2014 to 38 (0.80%) in 2022. Moreover, students reporting "Slightly competent" decreased from 1,712 (48.93%) to 1,851 (38.98%) across this same period. However, the net increase in positive (slightly or very competent) responses increased from 2,801 (80.05%) in 2014 to 4,168 (87.77%) in 2024, suggesting an increase of 7.72% in student confidence across this time.

The second measure analyzed was self-reported confidence to "participate as a contributing and integrated member of an interprofessional team and fully embrace the value of teamwork in patient care" (teamwork). The percentage of respondents who reported "Very competent" increased from 1,731 out of a total 3,499 respondents (49.47%) in 2014 to 3,084 out of a total 4,749 respondents (64.94%) in 2022 (Figure 2B). While the number of "Not at all competent" responses increased from 4 (0.11%) to 12 (0.25%) from 2014 to 2022, the number of respondents selecting "Slightly incompetent" (2014: 25 (0.71%); 2022: 31 (0.65%)), "Neither competent nor incompetent" (2014: 248 (7.09%); 2022: 246 (5.18%)), and "Slightly competent" (2014: 1,491 (42.61%); 2022: 1,376 (28.97%)) all decreased during this timeframe. These data summarize a change in positive responses from 3,222 (92.08%) in 2014 to 4,460 (93.91%) in 2024, suggesting a minor increase (1.83%) in student confidence across this time.

Respondents who did not participate in IPE activities were not precluded from answering these two confidence measure questions. Therefore, we stratified the normalized confidence ratings on the two measures by reported IPE participation. Students who self-reported participating in IPE activities had significantly higher confidence ratings in both patient handoff (IPE Yes: mean=1.20±0.79 SD; n=28,201; 95% CI=1.19-1.22; IPE No: mean=1.02±0.86 SD; n=8,300; 95% CI=1.00-1.04; p<0.001; Figure 2C) and teamwork (IPE Yes: mean=1.49±0.64 SD; n=28,201; 95% CI=1.48-1.51; IPE No: mean=1.32±0.76; n=8,300; 95% CI=1.30-1.35; p<0.001; Figure 2C). Together, these data support that IPE training leads to more confidence and competence in students with aspects of patient care and teamwork that can enhance patient safety and health outcomes.

**Figure 3:**
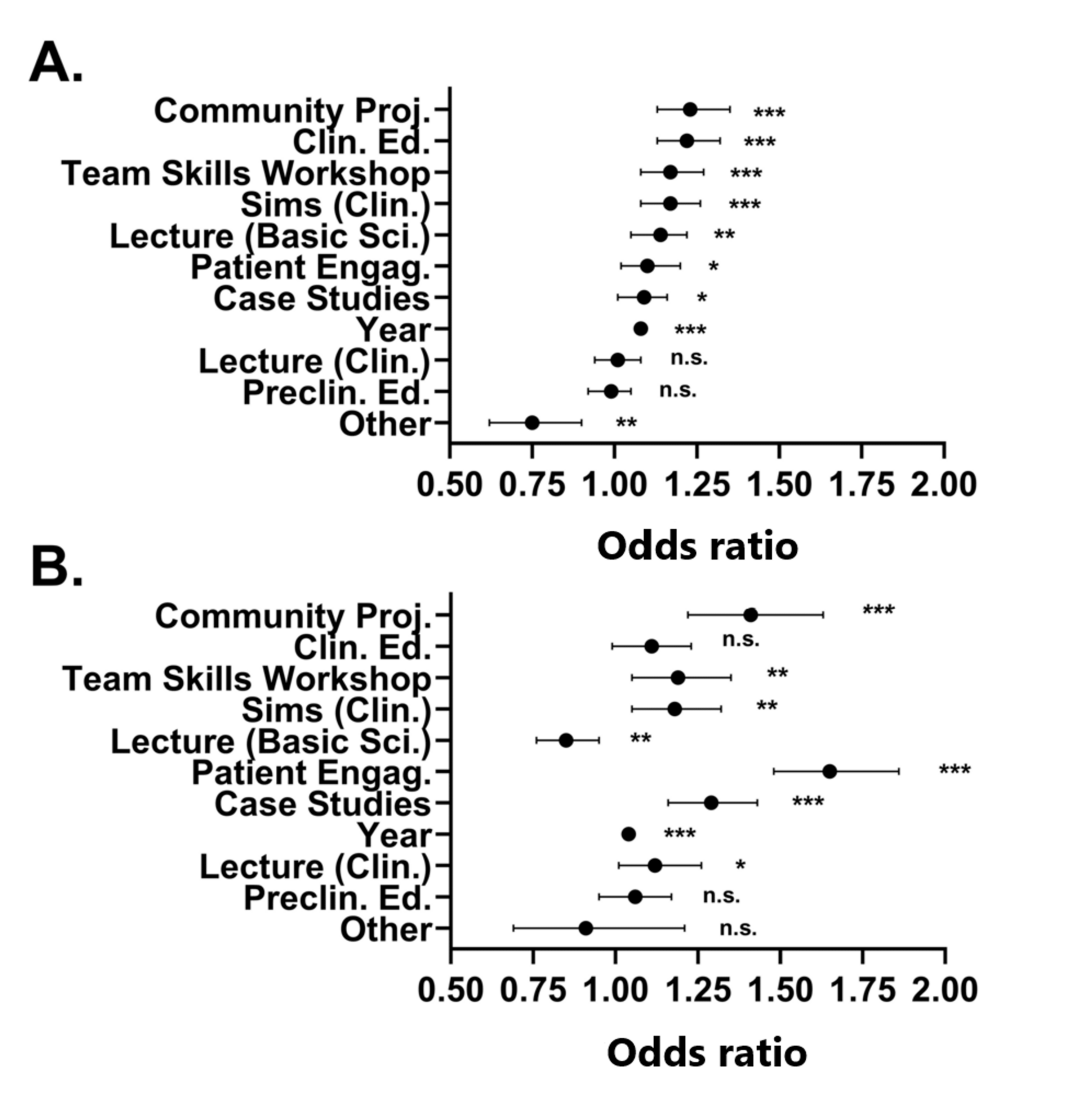
Forest plot of confidence in patient handoff and teamwork after IPE training activities (A) Odds ratio for self-reported confidence in patient handoff after the following IPE activities: "Community Projects or Service Learning" (Community Proj.), "Clinical Education" (Clin. Ed.), "Skills Training in Team Setting Workshops" (Team Skills Workshop), "Clinical Simulations" (Sims (Clin.)), "Lecture (Basic Science)" (Lecture (Basic Sci.), "Active Engagement with Patients (Rotations of Any Kind, Clinics, etc.)" (Patient Engag.), "Case Studies, Year, Lecture (Clinical Subject)" (Lecture (Clin.)), and "Pre-clinical Education" (Preclin. Ed.). (B) Odds ratio for self-reported confidence in teamwork after various IPE activities as in (A). *** indicates a p-value of <0.001; ** indicates a p-value of <0.01; * indicates a p-value of <0.05; and n.s. indicates a p-value of >0.05. IPE: interprofessional education

IPE activities are not standardized across colleges of osteopathic medicine, leading to variability in IPE training and potential student learning outcomes as reflected by these patient handoff and teamwork confidence measures [13]. In the AACOM graduating senior survey, students self-reported which IPE activities they participated in during their training. An odds ratio analysis (n=36,501) was performed to determine which IPE activity was correlated with the greatest competence responses for both patient handoffs and teamwork. For student competence in patient handoffs, "Community Projects or Service Learning" had the greatest odds ratio (Community Proj.: mean=1.23±1.05 (SEM); 95% CI=1.13-1.35; p<0.001; Figure 3A), followed closely by "Clinical Education" (Clin. Ed.: mean=1.22±1.04 (SEM); 95% CI=1.13-1.32; p<0.001; Figure 3A), "Skills Training in Team Setting Workshops" (Team Skills Workshops: mean=1.17±1.04 (SEM); 95% CI=1.08-1.27; p<0.001; Figure 3A), "Clinical Simulations" (Sims (Clin.): mean=1.17±1.04 (SEM); 95% CI=1.08-1.26; p<0.001; Figure 3A), and "Lecture (Basic Science)" (Lecture (Basic Sci.)): mean=1.14±1.04 (SEM); 95% CI=1.05-1.22; p=0.001; Figure 3A). Additionally, "Active Engagement with Patients (Rotations of Any Kind, Clinics, etc.)" (Patient Engag.: mean=1.10±1.04 (SEM); 95% CI=1.02-1.20; p=0.01; Figure 3A), "Patient-Centered Case Studies" (Case Studies: mean=1.09±1.04 (SEM); 95% CI=1.01-1.16; p=0.018; Figure 3A), and "Other" (Other: mean=0.75±1.10 (SEM); 95% CI=0.62-0.90; p=0.003; Figure 3A) were significantly correlated with greater confidence reports. Interestingly, "Lecture (Clinical Subject)" (Lecture (Clin.): mean=1.01±1.04 (SEM); 95% CI=0.94-1.08; p=0.814; Figure 3A) and "Pre-clinical Education" (Preclin. Ed.: mean=0.99; 95% CI=0.92-1.05; p=0.674; Figure 3A) were not statistically significant. We also analyzed student class year (year) as a parameter (Year: mean=1.08±1.01 (SEM); 95% CI=1.07-1.09; p<0.001; Figure 3A) which was significantly correlated to student confidence, consistent with our previous analyses (Figure 2).

For confidence scores in teamwork "Active Engagement with Patients (Rotations of Any Kind, Clinics, etc.)" (Patient Engag.: mean=1.65±1.06 (SEM); 95% CI=1.48-1.86; p<0.001; Figure 3B) had the greatest odds ratio, followed by "Community Projects or Service Learning" (Community Proj.: mean=1.41±1.08 (SEM); 95% CI=1.22-1.63; p<0.001; Figure 3B), "Patient-Centered Case Studies" (Case Studies: mean=1.29±1.05 (SEM); 95% CI=1.16-1.43; p<0.001; Figure 3B), "Skills Training in Team Setting Workshops" (Team Skills Workshops: mean=1.19±1.06 (SEM); 95% CI=1.05-1.35; p=0.005; Figure 3B), "Clinical Simulations" (Sims (Clin.): mean=1.18±1.06 (SEM); 95% CI=1.05-1.32; p<0.001; Figure 3B), "Lecture (Clinical Subject)" (Lecture (Clin.): mean=1.12±1.06 (SEM); 95% CI=1.01-1.26; p=0.037; Figure 3B), and "Lecture (Basic Science)" (Lecture (Basic Sci.): mean=0.85±1.06 (SEM); 95% CI=0.76-0.95; p=0.004; Figure 3B). "Clinical Education" (Clin. Ed.: mean=1.11±1.06 (SEM); 95% CI=0.99-1.23; p=0.078; Figure 3B), "Pre-clinical Education" (Preclin. Ed.: mean=1.06±1.05 (SEM); 95% CI=0.95-1.17; p=0.296; Figure 3B), and "Other" (Other: mean=0.91±1.15 (SEM); 95% CI=0.69-1.21; p=0.524; Figure 3B) were not significantly correlated to student self-report confidence in teamwork participation. When "Year" was analyzed as a parameter (Year: mean=1.04±1.01 (SEM); 95% CI=1.02-1.06; p<0.001; Figure 3B), it was significantly correlated to student confidence. These data indicate that not all IPE activities are equal in their ability to prepare students for IPE-related skills involving patient safety and teamwork.

## Discussion

Our findings show that, overall, students who participated in IPE between 2014 and 2022 had increased confidence in patient handoff and in their ability to work as an interprofessional healthcare team member. The factors correlated to increased confidence were identified using odds ratio analysis. For patient handoff activities, "Community Projects or Service Learning", "Clinical Education", "Team Skill Workshops", and "Clinical Simulations" had the greatest correlation. Interestingly, for confidence in ability to work as a member of a healthcare team, "Patient Engagement", "Community Projects or Service Learning", and "Case Studies" demonstrated the greatest correlation to self-reported confidence. Moreover, the "Community Projects or Service Learning" IPE training activity had a strong correlation with both patient handoff and teamwork confidence. Conversely, the "Pre-clinical Education"-based IPE training did not correlate significantly with confidence levels in either patient handoff or teamwork measures, and "Clinical Lectures" demonstrated either no correlation (patient handoff) or a mild correlation (teamwork). These data suggest that a diverse set of IPE training activities are required to improve student confidence outcomes; however, not all activities appear beneficial. The data presented will help guide programs towards the IPE activities that will be most impactful on student confidence.

IPE training is a critical component of medical education in the United States, and in 2019, the Health Professions Accreditors Collaborative released guidance on the curricular development of IPE to help standardize training [14]. However, there are other tools that can help with patient handoffs. Indeed, it was reported that failures in handoff could have been averted in 77% of cases by the use of a handoff tool [4]. One such handoff tool is the Illness severity, Patient summary, Action lists, Situational awareness and contingency planning, and Synthesis by receiver (I-PASS) mnemonic [15,16]. One study of I-PASS implementation in residency programs across 32 hospitals showed a 47% decrease in major and minor handoff-related reported adverse events [17]. Additionally, the Department of Defense (DoD) and the Agency for Healthcare Research and Quality (AHRQ) developed TeamSTEPPS™ as the national standard for team-based training in healthcare [18]. This toolkit has been demonstrated to improve care in both inpatient and outpatient settings [19,20]. Moreover, the integration of TeamSTEPPS™ into the IPE training curricula has been investigated and reported to be readily adaptable to meet the needs of the required IPE training, although large variability and lack of standardization are still observed [21]. Therefore, implementation of such tools early in physician training, especially in the setting of other IPE training activities such as participation in community projects or service learning events, could lead to improved patient safety.

Study limitations

Despite the COCA requiring IPE, only 78% of students reported participation in IPE for the AACOM 2022-2023 Graduating Seniors Survey [7]. This is down from 83% a year prior [8]. Additionally, there was a decrease in participation from nursing students (down to 69% from 76% in 2021), physician assistant students (down to 56% from 60% in 2021), and public health students (down to 12% from 17% in 2021) over these times [7,8]. Due to COCA requiring IPE, it is unclear why participation numbers are not greater. With divergent definitions and nomenclature, it is possible that many students aren't aware they are doing IPE or viewed it unfavorably and did not wish to self-report [22]. The lack of student self-reporting on IPE activity is an area requiring additional investigation. Additionally, investigations on barriers to participation in IPE activities in medical schools indicate that time constraints, scheduling conflicts, and communication barriers were the most significant detriments to IPE participation among medical students [23,24].

Another limitation of this study is the lack of specificity on which IPE activity would generate the greatest outcomes. There are a multitude of ways to implement a "Community Projects or Service Learning" IPE module in a curriculum, but best practices are not clear from the data analyzed in this study and require additional investigation.

## Conclusions

This study identified specific IPE activities that had a significant correlation with student confidence in patient handoff activities and in working as an interprofessional team member. Despite multiple studies showing benefit in IPE, various IPE training activities, such as "Pre-clinical Education", may not be as beneficial, thus undermining the goals of IPE. As team cooperation continues to be increasingly important to patient safety and outcomes, an IPE integrated curriculum would make a positive difference in patient safety outcomes when utilized in an effective manner.
